# Automated paleontology of repetitive DNA with REANNOTATE

**DOI:** 10.1186/1471-2164-9-614

**Published:** 2008-12-18

**Authors:** Vini Pereira

**Affiliations:** 1 Department of Life Sciences, Imperial College London, Silwood Park campus, Ascot, Berkshire SL5 7PY, UK; 2 Theoretical Systems Biology, Institute of Food Research, Norwich Research Park, Colney, Norwich NR4 7UA, UK

## Abstract

**Background:**

Dispersed repeats are a major component of eukaryotic genomes and drivers of genome evolution. Annotation of DNA sequences homologous to known repetitive elements has been mainly performed with the program REPEATMASKER. Sequences annotated by REPEATMASKER often correspond to fragments of repetitive elements resulting from the insertion of younger elements or other rearrangements. Although REPEATMASKER annotation is indispensable for studying genome biology, this annotation does not contain much information on the common origin of fossil fragments that share an insertion event, especially where clusters of nested insertions of repetitive elements have occurred.

**Results:**

Here I present REANNOTATE, a computational tool to process REPEATMASKER annotation for automated i) defragmentation of dispersed repetitive elements, ii) resolution of the temporal order of insertions in clusters of nested elements, and iii) estimating the age of the elements, if they have long terminal repeats. I have re-annotated the repetitive content of human chromosomes, providing evidence for a recent expansion of satellite repeats on the Y chromosome and, from the retroviral age distribution, for a higher rate of evolution on the Y relative to autosomes.

**Conclusion:**

REANNOTATE is ready to process existing annotation for automated evolutionary analysis of all types of complex repeats in any genome. The tool is freely available under the GPL at .

## Background

Repeats of high sequence complexity – mostly transposable elements (TEs) – account for a large portion of many eukaryotic genomes. In humans they comprise almost half of the (cytologically euchromatic) genome [1], and in some plants (e.g. maize) most of the DNA (>70%) is repetitive [2-4]. In addition to genome size evolution, repetitive sequences are fundamentally implicated in structural and functional genome evolution. Sequence similarity and complementarity form the basis of many biochemical reactions involving nucleic acids. Hence the occurrence of repeated sequences may mediate genome, epigenome and transcriptome interactions, such as chromosomal rearrangements [5-7], centromere [8] and telomere [9] function, and chromatin remodelling and gene silencing mediated by repeat-induced small RNAs [10-15]. In addition to the effects of sequence repetition, TE insertions may have sequence-specific phenotypic consequences. For instance, they encode regulatory signals that can potentially affect gene expression [16-19].

Given the importance of repetitive DNA sequences for genome structure and evolution, systematic annotation of repeats is essential for inferring biological organisation and function from genomic sequences. When large amounts of sequence data are analysed, automation of the annotation procedure is indispensable. REPEATMASKER[20] has become the default computational tool for automated repeat annotation. Despite the current indispensability and efficiency of REPEATMASKER in annotating genomic regions similar to known families of repeats, the REPEATMASKER annotation contains relatively little information on the origin and evolution of repeats. For example, if a given TE insertion is subsequently targeted by further insertions, the original TE sequence will be interrupted and fragmented by sequences of later origin. In such a situation REPEATMASKER may annotate multiple sequence similarity hits to the given TE family without establishing the common origin of these sequences [21-24], and no information on the temporal order of insertion of overlapping repetitive elements is obtained without human analysis. This situation is common as TEs are non-randomly distributed within genomes and are often 'nested', i.e. inserted into another TE. Nesting has been observed in diverse genomes across the eukaryotic kingdom [10,24-32]. Thus, post-processing is necessary to improve the biological interpretation of REPEATMASKER annotations (reviewed in ref. [33]).

Here I describe REANNOTATE (*R*epetitive *E*lement *re-annotation*), a computational tool for automated defragmentation and evolutionary analysis of (high complexity) repetitive DNA elements (mainly TEs). The term *re-annotation *reflects the use of REPEATMASKER annotation as input to REANNOTATE, and in this context it means neither the prediction of previously unannotated sequence features (similarity hits) nor the detection of false positives in the original annotation. Rather, it means adding "layers" of information that contain new *kinds *of inferences not present in the original annotation. REANNOTATE automatically generates up to three layers of re-annotation: *i) defragmentation*, via construction of repetitive element "models" consisting of sequence features originally annotated as separate similarity hits; *ii) order of insertion*, where TE models constructed in *(i) *overlap; and *iii) age*, for long terminal repeat (LTR)-elements in particular (dating of their insertion events is performed for structurally complete elements). In addition to annotation, REANNOTATE can output the sequences of defragmented repetitive element models, with appropriate gaps so that elements classified in a given family are all pairwise aligned to the family reference sequence; in this form they can easily be multiply aligned (which would be non-trivial with ungapped sequences if they have large indels relative to one another) and are ready for phylogenetic analysis.

REANNOTATE is ready to re-annotate existing REPEATMASKER annotation – either in its original format or as REPEATMASKER track tables from the UCSC Genome Browser web site . The first two layers of automated re-annotation can be visualised in the APOLLO genome browser [34], and in addition they can be combined with other kinds of annotation (e.g. non-repetitive genes), facilitating direct human analysis when required.

Among the functions performed by REANNOTATE, defragmentation of genomic sequence regions homologous to TEs has been previously addressed by other tools [22,24,35]. MATCHER[22] uses a dynamic programming algorithm to defragment TEs, but does not include biological constraints that can assist the defragmentation process. PLOTREP [35] is an interactive tool that assists manual defragmentation but cannot provide fully automated defragmentation necessary for genome scale analysis. Recently, TCF [24] has become available for automated identification and defragmentation of TE clusters, but TCF does not attempt to defragment all TEs – notably pieces of LTR-elements found in clusters, and fragments of TEs nested within another TE. REANNOTATE provides fully automated defragmentation of any kind of TE using biologically informed constraints. Importantly, REANNOTATE is able to defragment LTR-elements, which are often represented by separate query sequences for LTRs and internal regions, and to estimate the age of LTR-element insertion events. Other important differences between REANNOTATE and TCF include: *i) *TCF was developed for mammalian genomes and visualisation of annotation is limited to genomes available in the UCSC genome browser web site, whilst REANNOTATE is ready for the re-annotation of any genome and its visualisation (using APOLLO); *ii) *TCF effectively defragments only TEs that are interrupted by other previously characterised TE sequences, whilst REANNOTATE allows interruptions by any kind of sequence (e.g. unknown TEs) and also re-annotates complex repeats other than TEs; *iii) *parameters in the REANNOTATE defragmentation algorithm may be set by users to adapt the algorithm to the repetitive content of a particular genome; and *iv) *the annotation produced by REANNOTATE has been *validated *by comparison with manually curated annotation of sequences containing highly nested clusters of TEs. During the write-up of this paper, another tool, TENEST[36], has become available that has similar functionality to REANNOTATE. However, there are also important differences between REANNOTATE and TENEST: *i) *TENEST currently uses plant repeat databases, and is therefore designed to annotate plant genomes, whilst REANNOTATE is ready to annotate any genome. *ii) *TENEST itself manages the sequence similarity searches (using WUBLAST[37] and LALIGN [38]) against the repeat library, whilst REANNOTATE processes the similarity annotation produced by RepeatMasker. The similarity search is the most computationally expensive step in the annotation process, and because RepeatMasker annotation is already available for many genomic sequences, the re-annotation of such sequences is computationally cheaper with REannotate. *iii) *The visualisation method employed by REANNOTATE allows the repetitive DNA annotation to be combined with other kinds of genome annotation (see below).

In order to illustrate the kind of analysis that become possible with REANNOTATE, an application of whole chromosome re-annotation is provided for a human sex chromosome and two autosomes, providing analyses of *i) *repetitive element patterns of nesting (including evidence for recent expansion of satellite repeats on the Y chromosome), and *ii) *the age distribution of endogenous retroviruses.

REANNOTATE is open source and freely available under the GNU Public License at 

## Implementation

### Repetitive element model construction and re-annotation algorithm

#### A. Input *REPEATMASKER*annotation

**Figure 1:**
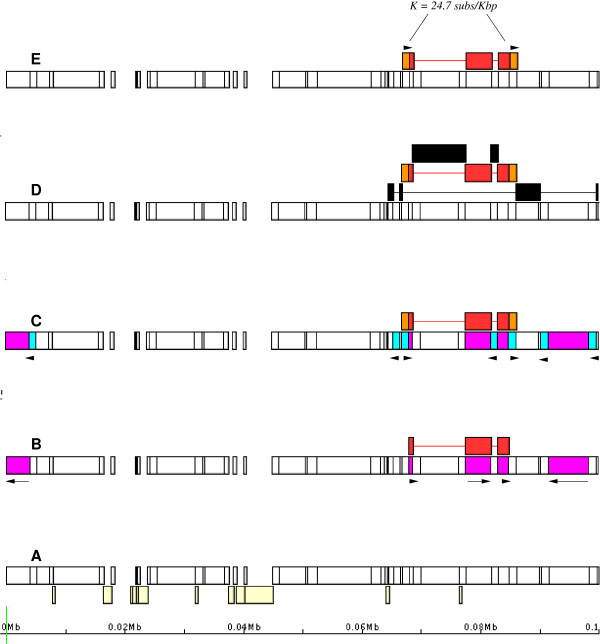
**Re-annotation algorithm**. A. Graphic representation of the input REPEATMASKER annotation of the first 100 Kb of genomic sequence [GENBANK:AF123535.1] around the *adh *gene of the maize cultivar *LH_82 *(this refers to the same maize sequence that was manually annotated in [45] and used to validate REANNOTATE's predictions in Results, Table 1, and Figure 3). B. Boxes highlighted in magenta on the bottom tier represent hits to the reference element PREM2_ZM_I (the internal region of an LTR-retrotransposon in REPBASE), of which the three innermost hits, shown again in red on the top tier united by a horizontal line, were defragmented by REANNOTATE into a repetitive element model. The black arrows show the orientation of the hits on the chromosome, and the three hits shown in red are colinear with the reference PREM2_ZM_I sequence. C. Boxes highlighted in blue on the bottom tier represent hits to the reference LTR sequence PREM2_ZM_LTR (in REPBASE). Above, two (single-hit) LTR models (shown in orange) flank an IR model (in red): these three models have been assembled into a higher-order model of an element of the PREM2_ZM family. D. The chromosomal span of the defragmented PREM2_ZM element (red and orange) is within the span of another element (bottom model in black); the PREM2_ZM element is inferred to have inserted into the element shown below it. Two other elements (black boxes on top tier) are inferred to have inserted into the PREM2_ZM element. E. Pairs of intra-element LTR sequences are output, aligned with CLUSTALW, and the number of point substitutions between them estimated.

REPEATMASKER annotation either in its original format or as a UCSC table is input to REANNOTATE. If this annotation reports similarity to *N *different reference repetitive elements, let *R *= {*r*^1^, ... *r*^*N*^} denote this library set of *N *reference elements. Here I call a *hit *to the reference element *r *a query sequence region homologous to *r *and with higher sequence similarity to *r *than to any other reference element in *R*, annotated by REPEATMASKER. As an example, a visual representation of the input annotation is given in Figure 1A.

#### B. Defragmentation of repetitive elements

REANNOTATE constructs repetitive element models assigned to the different families in the reference library *R*. A given reference element *r *defines a set of hits along the query sequence. REANNOTATE will search for subsets of hits to *r *that are to be defragmented into an element model if they satisfy the criteria:

(i) *Colinearity with the reference element*.

(ii) *Maximum span*.

Criterion (i) requires that the defragmented hits be in the same orientation on the query sequence, and that they match consecutive (though not necessarily contiguous) regions of the reference element *r*. (Note that along the query sequence these defragmented hits need not be consecutive, in the sense that there may be other hits to *r *nested between them.) An example is illustrated in Figure 1B. The reference element in question is PREM2_ZM_I (from REPBASE UPDATE), and hits to this reference element along the maize sequence are highlighted in magenta. REANNOTATE constructed a model defragmenting the three innermost hits to PREM2_ZM_I, shown in red, which respectively match nucleotide positions 1-787, 783-4958, and 4956-6864 along the reference PREM2_ZM_I sequence. Note that there is a small overlap between the matching coordinates along the reference sequence (5 nucleotide between the first two matches, and 3 nucleotides between the last two), which is an ostensible deviation from strict colinearity between the three hits on the maize chromosome and the reference sequence. This situation is common and is due to uncertainty about the ends of local alignments. REANNOTATE has a user-definable tolerance parameter ***ϵ ***in the requirement for colinearity between the element model and the reference element that allows for an overlap (*o*) between the matching coordinates of two defragmented hits along the reference sequence, if *o *≤ ***ϵ***. (Default ***ϵ ***= 40 nucleotides; if ***ϵ ***> *L*/10, where *L *is the length of a given reference sequence *r*, then the tolerance margin for that family is automatically set to *L/*10).

Criterion (ii) requires that the span (i.e. the query sequence length from the end of the first to the beginning of the last defragmented hit) of candidate repetitive element models do not exceed a (user-definable) length ***δ ***(default ***δ ***= 40 Kb).

For each reference family in the library *R*, REANNOTATE searches for candidate repetitive element models that satisfy (i) and (ii), but only constructs models that additionally satisfy:

(iii) *Uniqueness*.

(iv) *Maximum defragmentation*.

(v) *Recursive model nesting*.

Criterion (iii) requires that constructed models comprise mutually exclusive sets of hits.

If two candidate models are such that all the hits defragmented in one model are present in the other model that defragments a higher number of hits, then criterion (iv) requires that only the model defragmenting the maximum number of hits be constructed.

If two candidate models (assigned to the same family) comprise different sets of hits with at least one hit in common, then criterion (v) requires that the candidate model whose hits span the narrower region of the query sequence be constructed. If a hierarchy of such models exists then this pairwise criterion is repeated recursively. Additionally, if two candidate models (assigned to any families) are such that *a) *they comprise non-intersecting sets of hits and *b) *they span overlapping regions of the query sequence, then criterion (v) requires that they cannot both be constructed unless the span of one candidate model lies entirely within the span of the other.

#### Defragmentation of chromosomal elements matching multiple reference sequences

If, upon human inspection of either the original REPEATMASKER annotation or the automated re-annotation, the occurrence of fragments of a given repetitive element matching multiple reference sequences is detected, a user may supply as input to REANNOTATE a text file containing lists of "related" reference elements, so that hits to different reference elements within one such list may be considered for defragmentation into a repetitive element model. The use of this option was essential for the re-annotation and analysis of human endogenous retroviruses in Results; the ERV names equivalence lists are provided in Additional file 1.

#### C. Defragmentation of LTR-elements

The defragmentation procedure described in step *B *above applies to any high-complexity repetitive element. Both the LTR and the internal region (i.e. the sequence between the two LTRs of a complete element) of a given LTR-element family may be found as dispersed repeats. Fossil remains of a given insertion may contain only LTR sequence or only internal region (IR) sequence. REANNOTATE performs additional analysis of LTR-elements (i.e. LTR-retrotransposons and retroviruses) if the reference library contains separate entries for the LTR and IR sequences. Names of reference LTR and IR elements of the same family should be identical (unless a name equivalence list is input to REANNOTATE) apart from a suffix. Reference LTR names do not need a suffix, but they may be suffixed with either the string '-LTR' or '_LTR' (case-insensitive). Reference IR names should be suffixed with either '-int', '_int', '-I', or '_I' (case-insensitive). This is the naming convention used in REPBASE UPDATE for LTR-elements in most genomes, though currently with the notable exception of human/primate endogenous retroviruses (for the solution to this problem adopted in this study see below and Additional files 1 and 2).

Models of LTRs and IRs are separately constructed with the *Defragmentation *algorithm in step *B*. REANNOTATE then constructs higher-order models combining models of defragmented LTRs and defragmented IRs. Higher-order model construction proceeds with requirements analogous to those in step *B*, but now the colinearity criterion refers to the *LTR-IR-LTR *structure of a canonical LTR-element. An example is illustrated in Figure 1C. Recursive model nesting (as in B) is used to defragment LTR-element structures within structures. Where nested structures corresponding to the same family are identified, these are resolved by mapping the coordinates of the component LTR or IR fragments onto their respective reference sequences. A nested element should interrupt the nesting element. (If there is ambiguity in the sense that a given LTR could be paired with two different LTRs and IRs, a model of a 'complete' element is constructed with the two LTRs whose sequences are most similar to each other).

If a candidate higher-order model contains only one LTR and one IR model, a higher-order model for a truncated LTR-element is constructed only if the LTR and IR are separated on the query by less than a (user-definable) distance ***σ ***(REANNOTATE default ***σ ***= 15 Kb). Furthermore, truncated higher-order models are only constructed when the constituent LTR and IR models cannot be accommodated in a model of a 'complete' LTR-element.

REANNOTATE classifies LTR-element models as either *(i) *'complete', if they contain at least part of the sequence of *both *their original LTRs and of the IR; or *(ii) *'truncated', if they are not 'complete' and not a 'solo' LTR; or *(iii) *a 'solo' LTR, if a model contains only sequence corresponding to a single LTR, and if this is separated from the nearest LTR or IR model – of the same family and inserted in the same orientation – by a distance greater than ***σ***. (Note that the term 'solo LTR' is often used to mean an element that resulted from a deletion event that occurred via recombination between intra-element LTRs; such an event would preserve target site duplications (TSDs) flanking the original element, however REANNOTATE currently does not check for TSDs, and such a check would only be possible if the termini of the original element had not been truncated).

#### DNA rearrangements other than transposition

REANNOTATE will flag the possibility that LTR-element sequences have been involved in DNA rearrangements other than transposition of an entire element when an *IR-LTR-IR *structure is detected, i.e. two IR models flanking an LTR model of the same family, in the same orientation. Here "flanking" means that either *i) *the LTR model and the IR models are contiguous on the query sequence (within a tolerance margin ***ϵ***), and that neighbouring ends have no missing sequence (within ***ϵ***) – this excludes the possibility of a nested insertion of an IR or LTR of the same family being considered; or *ii) *the LTR and IR models are not contiguous on the query and they are not complete, but the length of the gaps between them equals the amount of sequence they are missing (within ***ϵ***) – this includes structures in which the *IR-LTR *boundaries have been obliterated (possibly prior to the re-arrangement), or structures with segments that are not homologous to the closest library sequence; or *(iii) *the structure is highly symmetrical, i.e. the two IR models are equidistant from the LTR model between them (within ***ϵ***), and provided that this separation on the query is less than ***σ***.

#### D. Inference of nesting order

After all hits to high-complexity repetitive elements have been defragmented into element models, any nested structures can be resolved by comparing the coordinate ranges of the models along the query sequence. If the span of a given model is contained within the span of another model, the former is classified as 'nested' in the latter (inferred to have inserted into the latter). An example is continued from step *C *and shown in Figure 1D.

REANNOTATE also provides an *optional *algorithm for the identification of 'truncated nesting': if one terminus of a given element model interrupts another model, the interrupting element may be classified as nested even if the interrupted element does not contain detectable sequence on both sides of the interrupting element. Truncated nesting is annotated only if there is no sequence missing from the the interrupting terminus of the interrupting element (so that a deletion spanning part of the truncated element and part of the interrupting element is not considered).

#### E. Dating of LTR-elements

For each (structurally) complete LTR-element model constructed, REANNOTATE outputs the gapped sequence (see below) of each intra-element LTR separately. REANNOTATE then generates automated intra-element LTR alignments using the CLUSTALW(2) [39] alignment program. The number of nucleotide substitutions per site (*K*) between intra-element LTR sequences (and its variance) is then estimated (Figure 1E) using the Kimura 2-parameter model [40]. If a rate of substitutions per site (*s*) is provided, the time elapsed since the insertion of a 'complete' LTR-element (*t*) is estimated as $t=\frac{K}{2s}$. In addition to the variance propagated from the estimation of *K*, the variance of the time estimate ($\sigma_{t}^{2}$) accounts for the fact that the accumulation of nucleotide substitutions occurs stochastically over time, which can be modeled as a Poisson process; the variance in *t *is then estimated as $\sigma_{t}^{2}=\frac{{\left(\sigma_{K}L\right)}^{2}+KL}{4{\left(sL\right)}^{2}}$, where *σ*_*K *_is the standard deviation of the estimate of *K*, and *L *is the length of the intra-element LTR alignment.

### Gapped sequence output

**Figure 2:**
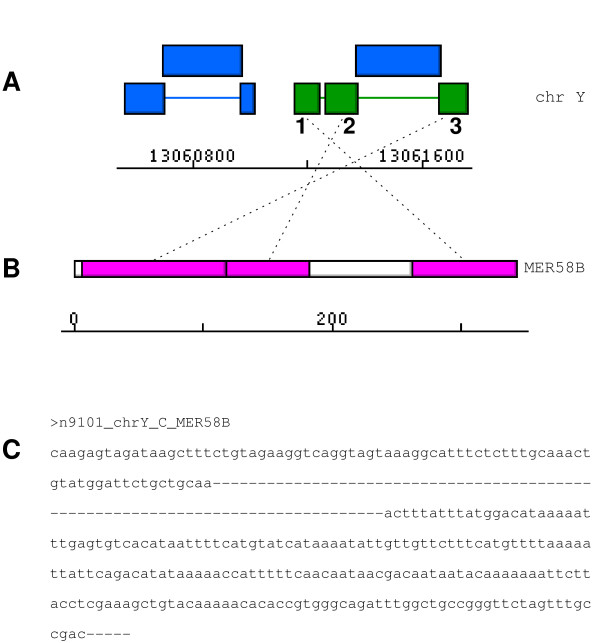
**Sequence output**. The element model shown in green (in A) defragmented three regions of human chromosome Y homologous to segments of the reference sequence of the DNA transposon family MER58B (CHESHIRE_B), shown in B. Hits marked 1 and 2 (in A) are separated on the chromosome by only 26 bp, but in the output model sequence (shown in C) their respective sequences are separated by an internal gap of length 79 – this is the distance along the reference MER58B separating its segments that are aligned to hits 1 and 2. In contrast, the sequences of hits 2 and 3 are output contiguously (without an intervening gap) because they match contiguous segments of MER58B – even though the corresponding chromosomal regions are separated by an ALUSX SINE insertion (blue box above the green model in A). The terminal gap in the model sequence is added to indicate that the annotated alignment of hit 3 ends five nucleotide positions short of the 5' terminus of the MER58B sequence.

Each repetitive element model constructed by REANNOTATE is associated with a given reference element *r*; each similarity hit defragmented into a given model is locally aligned to *r *by the sequence similarity search engine used with REPEATMASKER, either WUBLAST[37] or CROSS_MATCH[41]. For each model REANNOTATE outputs the chromosomal sequences (that have been locally aligned to *r*) of its hits, separated by gaps if necessary. The gaps refer to local alignment positions along *r*: in the output model sequence the gap length between two hits does not correspond to their distance along the chromosomal sequence, but rather to the distance between the hits' terminal alignment positions along *r*. Terminal gaps are also included if the hits to do not align as far as the termini of *r*. An example is given in Figure 2.

If REANNOTATE is run with the option to estimate the age of structurally complete LTR-element models then the CLUSTALW2 pairwise alignments of gapped intra-element LTR sequences are also output.

### Evaluation of REANNOTATE's predictions

Accuracy of the defragmentation layer of re-annotation was assessed by its sensitivity and specificity at the element level. A high sensitivity would indicate that most of the hits in the input REPEATMASKER annotation that correspond to fragmented repetitive elements in the manually annotated query sequence have been correctly assembled into multi-hit element models by REANNOTATE. A high specificity would indicate that few hits that are not part of fragmented elements have been incorrectly included into multi-hit element models. The sensitivity and specificity were respectively calculated according to the formulas $\frac{TP}{TP+FN}$ and $\frac{TN}{TN+FP}$, where *TP *(count of true positive predictions) is the number of hits correctly assembled into multi-hit repetitive element models; *FN *(count of false negatives) is the number of separate element models constructed by REANNOTATE that correspond to the same elements in the manual annotation; *TN *(count of true negatives) is the number of hits correctly modeled as single-hit elements; and *FP *(count of false positives) is the number of hits incorrectly assembled into multi-hit models. Accuracy of both the nesting structure and the time layers of re-annotation was calculated as the proportion of REANNOTATE predictions in agreement with manually curated annotation.

### Input to REANNOTATE

#### Analysis of maize and wheat sequences

Both the maize [GENBANK:AF123535.1] and wheat [GENBANK:AF459639.1] sequences were annotated with REPEATMASKER using a cutoff score of 200. Maize repeat sequences in the REPBASE UPDATE[42] repeatmaskerlibrary version 20050112 were used as a reference library, supplemented with sequences from the TIGR ZEA REPEAT DATABASE v3.0 [43]. Library elements CINFUL1_ZM and CINFUL2_ZM are closely related, and hits to these elements were considered together for defragmentation into element models. In the diploid wheat analysis the monocot library from the REPBASE UPDATE repeatmaskerlibrary version 20050523 was used.

#### Analysis of human and fly sequences

REPEATMASKER annotation was downloaded directly from the University of California at Santa Cruz (UCSC) Genome Browser web site .

Annotation of the human genome sequences was further processed with a custom script (Additional file 2) to suffix the names of reference LTR and IR elements for defragmentation. Additionally, a file with ERV reference name equivalence lists (Additional file 1) was input to REANNOTATE. This is because many HERV reference sequences in REPBASE UPDATE (which were used in the REPEATMASKER annotation) are closely related but may have disparate names, and for most ERV families the corresponding LTR and IR reference sequences have different names.

### Re-annotation output

The main annotation is output to a tab-delimited text file. As an example, the annotation of the maize sequence analysed here is given in Additional file 3, and its data fields are described in Additional file 4.

A copy of the input REPEATMASKER annotation is also output, with the original "ID" column replaced with identifiers of defragmented elements in the main REANNOTATE annotation file.

### Benchmarking of processing time

In order to illustrate the computational cost of re-annotation, I have benchmarked the CPU time used by REannotate for processing the entire *Arabidopsis thaliana *genome. REPEATMASKER annotation of the *A. thaliana *genome was downloaded from , and the corresponding genome sequence was downloaded from .

Running (under GNU/LINUX) on a 3.16 GHz INTEL E8500 processor, REannotate took:

• 157 CPU *seconds *to re-annotate the entire genome and output sequence. Out of these 157 seconds, 62 seconds were used by CLUSTALW2 to produce alignments. (19213 repetitive element models were constructed, 3619 nested insertions were inferred, and 294 intra-element LTR pairs were aligned).

• 107 CPU seconds (57 seconds used by CLUSTALW2) in total, to re-annotate the five A. thaliana chromosomes if the input RepeatMasker annotation was processed separately for each chromosome (i.e. the input annotation for each chromosome was stored in separate files).

• 195 CPU seconds (86 seconds used by CLUSTALW2) to re-annotate the entire genome when equivalence lists of reference repeats were used to defragment chromosomal elements matching multiple reference sequences. (Nine equivalence lists were used: 1. *athila4 athila4A athila4B athila4C*; 2. *athila8A athila8B*; 3. *athila6 athila athila2 athila0*; 4. *athila6A athila6B athila6*; 5. *athila athila5 athila2*; 6. *athila0 athila3*; 7. *atgp3 atgp5 atgp7*; 8. *atgp1 atgp2N*; 9. *ATREP3 ATREP4*). (18880 element models were constructed, 3673 nested insertions were inferred, and 393 intra-element LTR pairs were aligned).

Processing times will be much longer if many equivalence lists (such as those in Additional file 1, which exceed 100) are input to REannotate.

## Results and discussion

### Re-annotation

REANNOTATE creates up to three new 'layers' of repetitive DNA annotation over existing REPEATMASKER[20] annotation taken as input. The new layers of annotation are:

#### Layer 1 – Defragmentation

**Figure 3:**
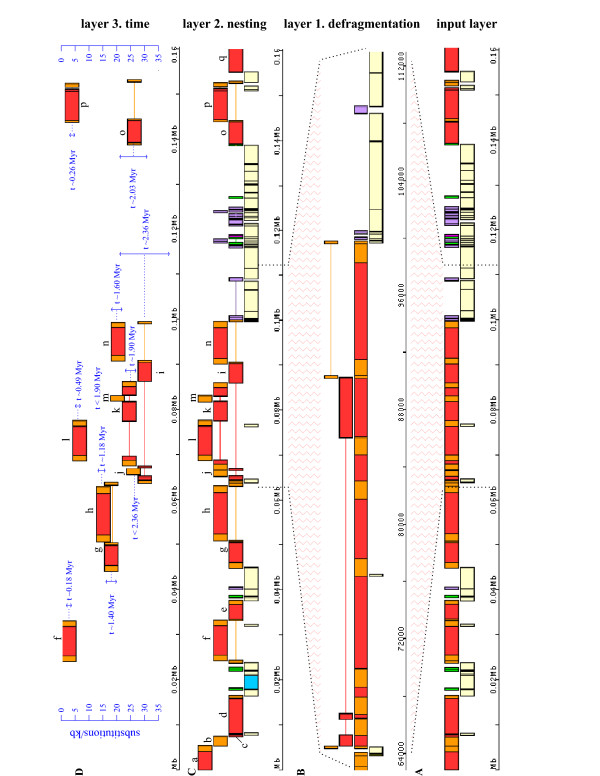
**Layers of re-annotation: maize adh1 locus. **A. Representation of the input REPEATMASKER annotation of 160 Kb of sequence around the adh1 locus of maize cultivar LH82. In the bottom tier pale yellow boxes correspond to un-masked sequence (no similarity to known repeats), dark vertical lines indicate low-complexity repeats. Boxes in top tier represent similarity hits to dispersed repeats, separated by vertical lines. Red boxes represent hits to the IR of LTR-elements, orange boxes LTRs, lilac boxes non-LTR retrotransposons, dark green boxes DNA transposons, and the pink box an unknown type of repeat. B. Detail of re-annotation layer 1: Defragmentation. The two bottom tiers represent a portion of the REPEATMASKER annotation shown in A, whilst boxes above indicate 3 IR hits (third tier from bottom) and 3 LTR hits (top tier) defragmented into a single model, and therefore inferred to share an insertion event. This element is labeled 'i' in C and in Table 1, and corresponds to element Victim in 45. (Three hits modeled as part of the same IR are linked by a red line, two hits modeled as part of the second LTR linked by an orange line). C. Re-annotation layer 2: Nesting Structure. Overlapping element models are shown in their order of insertion as resolved by REANNOTATE. (Figure created by rendering in APOLLO GFF annotation automatically generated by REANNOTATE). Letters label LTR-elements in Table 1 that were  annotated in 45. D. Re-annotation layer 3: Time. For 'complete' LTR-elements the number of substitutions (vertical scale) between intra-element LTRs and the time since insertion have been automatically estimated. Upper bounds on the ages of two solo LTRs (labeled 'j' and 'm') could be placed as the elements are inserted into complete LTR-elements. Double-headed arrows span two standard deviations around estimates of K. This figure may be compared to Figures 1 and 2 in 45.

After integration, the sequence of a repetitive element may become fragmented by subsequent insertion/deletion events or other rearrangements. REANNOTATE generates models of repetitive element insertion events into an ancestral state of a query genomic sequence, in order to identify query sequence regions that originated in the same insertion event and that correspond to (possibly) multiple hits in the input REPEATMASKER annotation. Therefore for a given re-annotated query sequence the number of TE hits in the REPEATMASKER annotation is usually greater than the total number of repetitive element models obtained from the defragmentation of hits performed by REANNOTATE (the inferred number of insertion events). Figure 3A shows a visual representation of the REPEATMASKER annotation of multiple similarity hits to TEs along a DNA sequence. Figure 3B shows, as an example, the re-annotation of 6 similarity hits that were defragmented into the same TE model.

Fragmentation of repetitive element sequences in the REPEATMASKER annotation may also result from divergent or chimeric elements relative to the reference sequences used in the similarity searches, or from matches to closely related sequences in the reference library, rather than sequence evolution of a repetitive element since its integration into the genome. REANNOTATE provides a facility to defragment hits in this situation as well (see section "Defragmentation of chromosomal elements matching multiple reference sequences" in Implementation). Additionally, DNA re-arrangements other than transposition of an entire element (e.g. segmental duplication) may occur involving repetitive elements after their integration; in this situation a single insertion model is not adequate to describe all of a query sequence that is homologous to a given repetitive element in the re-arranged region. Currently for LTR-elements (only) REANNOTATE checks for the possibility of re-arrangements that result in multiplication of segments of elements, and generates models of such re-arrangements (see below and Implementation).

REPEATMASKER annotation implicitly contains the chromosomal *distribution of sequence similarity hits *to dispersed repeats along the query sequence. This first layer of re-annotation consequently contains the chromosomal *distribution of repetitive element integration events*.

### Layer 2 – Nesting Structure

As the genomic distribution of repetitive elements is not random, families of elements may vary in their distribution patterns, which may reflect biases towards chromatin states or proximity to particular classes of genes or other sequence elements [44]. Clustering of repeats occurs not only at the chromosomal level, but also at smaller scales. In eukaryotic genomes, repeat deserts are commonly punctuated by regions of high repeat density, where subsequent insertions have occurred into previous repetitive element insertions ('nested' elements) [10,24-32]. Building upon the insertion models of the *Defragmentation *layer of re-annotation, REANNOTATE resolves any nested structures among the repetitive elements identified. Figure 3C shows resolved clusters of re-annotated TEs. The temporal *order of insertion events in nested structures *is reflected by the nesting level of each element model.

#### Layer 3 – Time

The direct, long terminal repeats (LTRs) of any individual LTR-retrotransposon or retrovirus are created from the same parent template, and are therefore identical at the time the element integrates into the host molecule. Nucleotide substitutions in either of the two intra-element LTR sequences can accumulate as time passes, so that their sequence divergence works as a molecular timer, set to zero at the time of integration [45]. REANNOTATE can generate automated intra-element LTR alignments and then estimate the number of nucleotide substitutions per site (*K*) that have occurred between the intra-element LTR of a given element since its insertion event. Estimates of *K *are a relative measure of time; but if the user can provide a rate of nucleotide substitution, estimates of *K *can be converted to estimates of absolute ages of (structurally) 'complete' LTR-elements. (Here by 'complete' I refer to elements for which at least a portion of each LTR is still detectable). Although direct dating of insertions is only available for 'complete' LTR-elements (and made possible by the previous *Defragmentation *layer of re-annotation), in nested structures bounds on the ages of other kinds of sequence elements can be estimated by using the age estimate for an overlapping LTR-element. If a 'complete' LTR-element is found in a nested cluster of repetitive elements, the previous *Nesting Structure *layer of re-annotation allows the placement of *i) *an upper bound on the age of any elements nested within, and *ii) *a lower bound on the age of any elements nesting, the LTR-element. Figure 3D shows examples of both direct dating of insertion events and indirectly using the nesting structure. Additionally, indirect dating of host molecules is possible in cases such as *i) *segmental duplications with a differential content of, and *ii) *haplotypes bearing insertional polymorphism for, 'complete' LTR-elements. Here a paleontological analogy is particularly apt, as the dating of both the molecular fossil and its sequence context are interrelated, just as real fossils can be dated by either absolute (e.g. radiocarbon) or relative (e.g. stratigraphic) methods. In eukaryotic genomes nesting of repetitive elements (see Background) and insertional polymorphisms [44,46-50], for example] are common – in plants particularly for LTR-elements.

This layer of re-annotation implicitly contains the *age distribution *of 'complete' LTR-elements, and bounds on the ages of other kinds of sequences that may be found overlapping LTR-elements.

#### Visualisation of re-annotation

In addition to the main re-annotation output to tab-delimited text files, for visualisation and human analysis of the automated annotation REANNOTATE generates a General Feature Format (GFF) annotation file. The GFF annotation can be visualised (using a configuration file distributed with REANNOTATE) in the APOLLO genome browser [34]. Figure 3C is taken from a screenshot of such a visualisation. The GFF output can be combined with any other kinds of annotation of the query sequences that are available in the GFF format.

### Sequence output

REANNOTATE retrieves (from the query) and assembles the sequence of all repetitive element models constructed. For each element model, it outputs the sequences of all the fossil fragments associated with a given model without intervening (unrelated) regions of the query sequence (Figure 2). Each pair of neighbouring fragment sequences is either *i) *output contiguously if they match contiguous segments of the reference sequence, or *ii) *output separated by a gap of length equal to distance between the respective matching segments of the reference sequence (see Figure 2 and Implementation). Thus these gapped sequences reflect the element insertion model and exclude the sequence of subsequent insertions into the element. For each 'family' of repeats (where 'family' refers to the set of elements in the query sequence that share a closest homologue in the reference library): *gapped element sequences are all aligned relative to the reference sequence*. Alignment of all the elements within a family, whose sequences may contain large indels relative to one another, is a powerful feature for the evolutionary (phylogenetic) analysis of repetitive elements.

### Validation: nested transposable elements in cereal genomes

**Figure 4:**
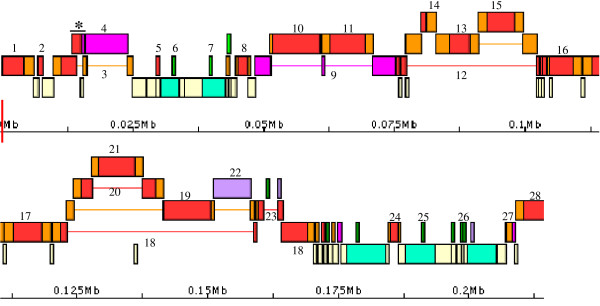
**Re-annotation of highly nested TEs in the diploid wheat genome**. Re-annotation of repeats in a 215 Kb region of *Triticum monococcum *chromosome 5A^*m*^. Numbers label elements listed in Table 1. Colour scheme follows Figure 3 (except that thin green boxes are specifically MITEs, and on the bottom tier unique genes are shown as light blue boxes). Label "18" appears twice and corresponds to element *Sabrina_G1-1 *annotated in [51] (Fig. 1 in this reference may be compared with this figure); REANNOTATE constructed two separate models because when run with default parameters the maximum span of a model is 40 Kb, which is exceeded by the chromosomal span of *Sabrina_G1-1*. Element models marked with a "*" above a horizontal bar were annotated as part of element *Sabrina_F2-2 *in [51], which corresponds to label "3" in this figure (see section 'Limitations and scope for development'). This figure was rendered in APOLLO from a GFF annotation file generated by REANNOTATE.

In order to validate annotation and evolutionary analysis generated by REANNOTATE, I have re-annotated repetitive elements in maize and wheat sequences, and then compared their automated re-annotation with published human annotation of these sequences. The term 'molecular paleontology' was coined by SanMiguel *et al*. [45], who produced manual annotation of nested clusters of LTR-retrotransposons within 240 kb around the *adh1 *locus of a particular maize cultivar and inferred a doubling of the maize genome size due to LTR-retrotransposon activity over a period of three million years – an inference that was possible by dating (using intra-element LTR sequence divergence) insertion events. Approximately 160 Kb of contiguous sequence and annotation for this locus are available. Detailed annotation of highly nested clusters of LTR-retrotransposons (including age estimates for complete elements) and other types of TEs is also available for a 215 Kb segment of chromosome 5A^*m *^of the diploid wheat *Triticum monococcum *[51]. Both the 160 Kb- and 215 Kb-long maize and diploid wheat sequences were annotated with REPEATMASKER and re-annotated with REANNOTATE (see Implementation for details). The automated re-annotation and evolutionary analyses are illustrated in Figure 3 (maize) and Figure 4 (wheat), and compared to the manually curated annotation in Table 1.

**Table 1:** Comparison between automated and human annotation of TEs

	repeat^*a*^		hits^*b*^	nests^*c*^		*K *± s.d. (× 10^3^)^*d*^		time ± s.d. (Mya)^*e*^		type
a	*Ji-6*	PREM2_ZM	2	*2*	2	...	-	...	-	LTR

b	*Tekay*	TEKAY_ZM	1	*1*	1	...	-	...	-	LTR

c	*Rle*	REINA	1	*0*	0	-	-	-	-	LTR

d	*Cinful-2*	CINFUL2_ZM	2	*0*	0	-	-	-	-	LTR

e	*Milt*	00081	3	*0*	0	*20.3 ± 5.5*	>2.4 ± 1.4	*1.56 ± 0.42*	> .18 ± .15	LTR

	*	00081	1	*0*	0	-	-	-	-	LTR

f	*Opie-2*	OPIE2_ZM	3	*1*	1	*2.4 ± 1.6*	2.4 ± 1.4	*0.18 ± 0.11*	0.18 ± 0.15	LTR

g	*Fourf*	00098	5	*0*	0	*18.1 ± 4.1*	18.2 ± 4.1	*1.39 ± 0.32*	1.40 ± 0.44	LTR

h	*Huck-2*	HUCK1	3	*1*	1	*12.3 ± 2.9*	15.3 ± 3.1	*0.95 ± 0.22*	1.18 ± 0.34	LTR

i	*Victim*	00093	6	*0*	0	*31.4 ± 19*	30.7 ± 18	*2.42 ± 1.44*	2.36 ± 1.92	LTR

j	*Ji-2*	PREM2_ZM	1	*1*	1	-	< 31 ± 18	-	< 2.4 ± 1.9	LTR

k	*Ji-3*	PREM2_ZM	5	*1*	1	*24.2 ± 4.8*	24.7 ± 4.7	*1.86 ± 0.37*	1.90 ± 0.51	LTR

l	*Opie-3*	OPIE2_ZM	3	*2*	2	*6.4 ± 2.3*	6.4 ± 2.3	*0.49 ± 0.18*	0.49 ± 0.25	LTR

m	*Ji-5*	PREM2_ZM	1	*2*	2	-	< 25 ± 5	-	< 1.9 ± 0.5	LTR

n	*Ji-4*	PREM2_ZM	3	*1*	1	*21.1 ± 4.2*	20.8 ± 4.1	*1.62 ± 0.32*	1.60 ± 0.44	LTR

o	*Reina*	REINA	4	*0*	0	*27.0 ± 9.8*	26.4 ± 9.4	*2.08 ± 0.75*	2.03 ± 1.02	LTR

p	*Cinful-1*	CINFUL1/2_ZM	4	*1*	1	*3.4 ± 2.4*	3.4 ± 2.4	*0.26 ± 0.18*	0.26 ± 0.26	LTR

q	*Kake-1*	00243	2	*1*	1	...	-	...	-	LTR

1	*Angela_F2-2*	ANGELA1_TM	2	*1*^†^	0	-	-	-	-	LTR

2	*RIRE2 (rice)*	SABRINA2_TM	1	*0*	0	-	-	-	-	LTR

3	*SabrinaF_2-2*	SABRINA2_TM	4	*0*	0	*25.9 ± 4.2*	26.6 ± 4.2	*1.99 ± 0.32*	2.04 ± 0.46	LTR

		SABRINA3_TM	1	-	1	-	< 27 ± 4	-	< 2.0 ± 0.5	LTR

		SABRINA_HV	1	-	1	-	< 27 ± 4	-	< 2.0 ± 0.5	LTR

4	*Nusif_F2-1*	NUSIF1_TM	1	*1*	1	-	< 27 ± 4	-	< 2.0 ± 0.5	LTR

5	*RIRE2 (rice)*	RIRE2	1	*0*	0	-	-	-	-	LTR

6	*MITE 1-4*	THALOS_HV	1	*0*	0	-	-	-	-	MITE

7	*MITE 2-5*	TREP220	1	*0*	0	-	-	-	-	MITE

8	*Veju_F2-1*	VEJU1_TM	3	*0*	0	*10.8 ± 5.5*	10.8 ± 5.4	*0.83 ± 0.42*	0.83 ± 0.59	LTR

9	*Claudia_F2-1*	CLAUDIA1_TM	3	*0*	0	-	> 41 ± 6	-	> 3.2 ± 0.6	LTR

10	*Latidu F2-1*	LATIDU2_TM	3	*1*	1	*13.3 ± 5.3*	13.1 ± 5.4	*1.01 ± 0.41*	1.01 ± 0.58	LTR

11	*Wham F2-1*	WHAM3_TM	3	*1*	1	*40.6 ± 5.6*	41.4 ± 5.6	*3.12 ± 0.43*	3.18 ± 0.60	LTR

12	*Fatima_F2-1*	FATIMA_TM	6	*0*	0	-	> 31 ± 4	-	> 2.4 ± 0.4	LTR

13	*Sukkula_F2-1*	SUKKULA3_TM	1	*1*	1	*29.9 ± 2.6*	-	*2.30 ± 0.20*	-	LTR

		SUKKULA3_TM	4	*1*	1	*29.9 ± 2.6*	30.7 ± 3.5	*2.30 ± 0.20*	2.36 ± 0.37	LTR

14	*Angela_F2-3*	ANGELA1_TM	2	*2*	2	-	< 31 ± 4	-	< 2.4 ± 0.4	LTR

15	*Angela_F2-1*	ANGELA1_TM	3	*2*	2	*19.9 ± 3.4*	19.9 ± 3.4	*1.53 ± 0.26*	1.53 ± 0.37	LTR

16	*Sabrina_F2-1*	SABRINA3_TM	2	*0*	0	-	-	-	-	LTR

17	*Wis_F2-1*	WIS4_TM	3	*0*	0	*58.1 ± 6.0*	57.0 ± 6.0	*4.47 ± 0.46*	4.38 ± 0.64	LTR

18	*Sabrina_G1-1*	SABRINA1_TM	3	*0*	0	*55.8 ± 6.1*	> 39 ± 5	*4.29 ± 0.47*	> 3.0 ± 0.6	LTR

		SABRINA1_TM	3	*0*	0	*55.8 ± 6.1*	-	*4.29 ± 0.47*	-	LTR

19	*Wham_G1-2*	WHAM2_TM	5	*1*	1	*39.1 ± 5.5*	39.1 ± 5.4	*3.01 ± 0.42*	3.01 ± 0.58	LTR

20	*Sabrina_G1-2*	SABRINA2_TM	4	*2*	2	*34.7 ± 4.8*	35.9 ± 4.9	*2.67 ± 0.37*	2.76 ± 0.52	LTR

21	*Wham_G1-1*	WHAM1_TM	3	*3*	3	*32.2 ± 4.9*	31.6 ± 4.8	*2.48 ± 0.38*	2.43 ± 0.52	LTR

22	*Miuse_G1-1*	MIUSE1_TM	1	*2*	2	-	< 39 ± 5	-	< 3.0 ± 0.6	LINE

23	*Latidu_G1-1*	LATIDU2_TM	3	*1*	1	-	-	-	-	LTR

24	*Eway_G1-1*^‡^	EWAY1_TM	3	*0*	0	*0*^‡^	73.1 ± 18	*0*^‡^	5.62 ± 1.87	LTR

25	*MITE 4A-10*	TREP216	1	*0*	0	-	-	-	-	MITE

26	*MITE 4A-4B*	TREP216	1	*0*	0	-	-	-	-	MITE

27	*Barbara*	BARBARA_TM	2	*0*	0	-	-	-	-	LTR

28	*Angela_G1-1*	ANGELA6_TM	2	*0*	1^†^	-	-	-	-	LTR

Manual annotation results of maize [45] and diploid wheat [51] sequences are shown in *italics*. REANNOTATE results are shown in regular font style. Only elements spanning sequences that were annotated as TEs both in the manual annotation and in the input (REPEATMASKER) to the automated re-annotation are listed. In the first column letters indicate maize elements and correspond to labels in Figure 3C, numbers indicate wheat elements and labels in Figure 4.

^*a *^Uppercase names correspond to reference element sequences in REPBASE UPDATE (RU), numbers correspond to reference sequences in the TIGR ZEA REPEAT DATABASE. Rows without an entry for the manually annotated repeat name indicate that REANNOTATE constructed multiple models (one model per row) corresponding to a single element in the manual annotation: for instance, *Sabrina_F2-2 *corresponds to three automated models, a result due to the fact that (parts of) different RU reference elements, SABRINA2_TM, SABRINA3_TM and SABRINA_HV are closely related, and were best matches (annotated by REPEATMASKER) to different segments of *Sabrina_F2-2*.

^*b *^Number of similarity hits reported by REPEATMASKER that were defragmented into a single element model by REANNOTATE.

^*c *^Number of repetitive elements nesting a given element. (^†^) The first wheat element listed was annotated in [51] to be inserted into a TE sequence with no detectable similarity to reference elements in RU (absent form the input REPEATMASKER annotation); the last wheat element was annotated by REANNOTATE to be interrupting a fragment of an element homologous to CLAUDIA1_TM, which is not present in the manual annotation.

^*d *^Estimated number of nucleotide substitutions between intra-element LTRs. (*) REANNOTATE did not date *Milt *because the 3' LTR is in inverse orientation relative to the rest of the element: an element model was built including the *Milt *5' LTR and internal region, and another model for the 3' LTR. (^‡^) *Eway G1-1 *was originally annotated as having identical LTRs, but they are in fact quite divergent. (...) Elements *Ji-6*, *Tekay *and *Kake-1 *were dated in the original annotation, but these elements are truncated at the ends of the available 160 Kb of contiguous sequence re-annotated here.

^*e *^Estimated time of insertion (million years ago), obtained with the substitution rate for the *adh *loci of grasses [66]. The standard deviations computed by REANNOTATE are larger than in the manual annotation: in the latter the variance in time was propagated from the variance in *K*, whilst REANNOTATE additionally accounts for the Poisson variance (stochasticity) in the accumulation of nucleotide substitutions.

Although the analyses above focused on the annotation of LTR-elements, they also validate the automated re-annotation of other kinds of repetitive elements and were chosen for two main reasons: *i) *Models of LTR-elements in the first (Defragmentation) layer of re-annotation are more complex than models of other repeats. LTR-element models involve defragmentation of LTRs (which are themselves repetitive elements) and defragmentation of the internal regions (which are also repetitive) using the same algorithm as for other repeats, and an additional algorithm to defragment LTRs and internal regions modeled as parts of the structure of the same element (see Implementation). Therefore validation of the automated defragmentation of LTR-elements can be extended to other repetitive elements. The second layer (*Nesting Structure*) of re-annotation builds on the first layer and uses the same algorithm for all elements. Finally, the third layer (*Time*) of re-annotation is directly applicable only to (structurally) complete LTR-elements (though secondary inferences can be made for other kinds of elements if they overlap with a complete LTR-element). Note that results for the Time layer are obtained independently from any information on *Nesting Structure *(except for the secondary inferences of bounds on the ages of overlapping elements). The *Time *layer annotation of the LTR-elements in the maize and wheat sequences analysed is completely consistent with the *Nesting Structure *annotation (Figures 3 and 4, and Table 1), adding support to the method for dating LTR-elements. Consistency between nesting structure and age estimates (obtained from a method different from the one used here) has also been shown for mammalian TEs using TCF [24], suggesting that this result is a general feature of the molecular paleontology of TEs. *ii) *For the maize and wheat sequences analysed, manually curated annotation of transposable elements was available that contained detailed information on the nesting order of repeats and dating of LTR-elements, providing a standard for comparing automated inferences against.

Here I compared (Table 1) only TE sequences that are present both in the manual and in the REPEATMASKER annotation input to REANNOTATE, because only those are relevant for evaluating REANNOTATE's inferences. REANNOTATE's predictions in the three layers of re-annotation achieved excellent accuracy relative to manual annotation, with results shown in Table 2 (see Implementation for calculation). The actual accuracy of predictions may be higher, as for some of the discrepancies the correct prediction may be attributed, upon inspection, to the automated re-annotation, despite the generally high quality of the human-curated annotation. For instance, the given accuracy of the *Time *layer predictions for the wheat sequence was obtained from 10 correct predictions out of 11 complete LTR-elements dated (90.9%). The one disagreement refers to the element *Eway_G1-1 *(Table 1) that was originally misannotated as having identical intra-element LTRs; the LTR sequences are actually quite divergent and REANNOTATE predicts the element to be the oldest of all complete LTR-elements found in the analysed query sequence.

**Table 2:** Accuracy of REANNOTATE's inferences relative to manual annotation

	Defragmentation		Nesting	Time
	Sensitivity	Specificity		
maize (AF123535.1)	97.8%	100.0%	100.0%	100.0%
wheat (AF459639.1)	96.0%	100.0%	93.3%	90.9%

Accuracy of predictions in the Defragmentation layer of re-annotation is given by their sensitivity and specificity according to the formulas $\frac{TP}{TP+FN}$ and $\frac{TN}{TN+FP}$ respectively, where *TP *is the count of true positives, *TN *true negatives, *FP *false positives, and *FN *the count of false negatives (see Implementation) relative to the manual annotations. Here a 'prediction' refers to a sequence similarity hit reported by REPEATMASKER that has been defragmented into a TE model by REANNOTATE. For the Nesting Structure and Time layers accuracy is given as the proportion of predictions in agreement with the original manual annotation [45,51].

### Repetitive DNA rearrangements other than transposition

**Figure 5:**
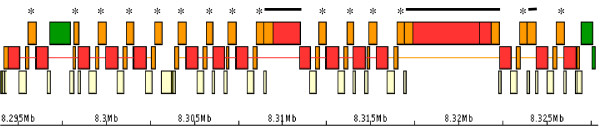
**Segmental multiplication within a TE cluster in the fly genome**. Re-annotation of a cluster of repeats in the *Drosophila melanogaster *chromosome arm 3R. The scale shows chromosomal coordinates (Release 3.1 genome sequence). Visualisation scheme as in Figure 3, except that "element" models – displayed as boxes united by horizontal lines – no longer indicate sequences sharing an insertion (transposition) event; here a model indicates sequences that resulted from segmental multiplication subsequent to an original insertion. Note the high periodicity of the arrangement. The LTR-elements displayed immediately above the bottom tier all belong to the COPIA2 family, the sequences marked with a '*' are all INVADER1 LTRs, and the ones marked with a black bar are MICROPIA elements. REANNOTATE infers that the COPIA2 sequences have been involved in DNA rearrangements other than transposition of an entire element. It is likely that the INVADER1 LTR was inserted in a COPIA2 LTR prior to the multiplication of the latter. The green box to the left indicates (subsequent) insertion of a PROTOP_A element, and the ones on the right S-elements. (All family names given as in the RU database). This figure was rendered in APOLLO from a GFF annotation file generated by REANNOTATE, and it may be compared with Figure 2 in ref. [32].

In order to demonstrate that REANNOTATE can correctly detect and annotate DNA re-arrangement events (involving TE sequences) that have occurred after integration, I have re-annotated a region of the *D. melanogaster *genome that has previously been shown to contain a large number of TE fragments that have arisen by the joint effects of integration and duplication [32]. This region lies between the *Hsp70 *genes on chromosome arm 3R and is denoted in ref. [52] as *NEST_FBti0020655 *(Release 3), and in ref. [32] as "high density region 16 (HDR16)" (Release 4). The re-annotation of *NEST_FBti0020655 *is shown in Figure 5, which can be compared to Figure 2 in ref. [32]. This serves to illustrate an additional type of inference that can be generated by REANNOTATE: the identification of segmental duplications contained in dispersed repeats (currently for LTR-elements only). Such a rearrangement could result from tandem duplication of a segment of an element, or from inter-element recombination events (e.g. between LTRs). Note that here REannotate attempts to construct an explicit model of sequence duplication to distinguish the origin of the repetitive sequences from independent transposition events.

The periodic structure of the LTR-element sequences in Figure 5 strongly suggests tandem multiplication. The multiple similarity hits (annotated by REPEATMASKER) to LTR-elements (in which other repeats are nested) in the structure (inferred by REANNOTATE) all belong to the same family (COPIA2), periodically map to the same regions of the reference sequence, and were re-annotated as elements that have been involved in a DNA rearrangement other than transposition. The 'solo' LTRs re-annotated as *nested in *the COPIA2 sequences all belong to the same family (INVADER1), they are also periodically arrayed (with the same spacing as the nesting sequences), and are inserted at the same position within the reference COPIA2 LTR.

Hence it is evident that the INVADER1 LTR was inserted in the COPIA2 LTR prior to tandem multiplication. Arrangements of the kind found in the *D. melanogaster *cluster *NEST_FBti0020655*/HDR16 (Figure 5) have also been reported for LTR-elements in yeast [53], for human ERVs [54], and have been detected with REANNOTATE in the *Arabidopsis thaliana *genome (data not shown). Thus it is possible that expansion of clusters of TE sequences via mechanisms in addition to transposition is a common phenomenon in eukaryotic genomes [32].

Although prediction of DNA rearrangements other than transposition is under-annotated by REANNOTATE (only certain kinds of rearrangement are currently detected – see Implementation), these annotations have value in *i) *cautioning the user against the validity of dating LTR-elements that may have been involved in post-integration recombination events; and *ii) *marking structures with unusual features for human inspection of their annotation.

### Genome Paleontology

The advent of automated defragmentation and sequence analysis of fossil fragments of dispersed repeats makes possible the study of the evolutionary dynamics of these elements (and their host molecules) at the scale of entire chromosomes or genomes [44,54].

In order to further illustrate evolutionary analyses that become possible with REANNOTATE, here I highlight the re-annotation of dispersed repeats in a human sex chromosome (Y) and in two autosomes (chromosomes 2 and 1).

#### Nesting of repeats in the human genome

Nesting patterns and counts of insertion events of repeats re-annotated on human chromosomes Y and 2 are summarised in Table 3. One striking result is the scarcity of TE insertions nested in satellite repeats, especially on chromosome Y. Even though, from these data, the possibility of functional constraint on satellite arrays cannot be ruled out, it is plausible that this result reflects a recent expansion of satellite repeats on human chromosome Y. Taking chromosome 2 as an example, not only the density of TE insertions into satellite sequence is almost four times that on the Y chromosome, but also there are ten times as many satellite repeats on the Y as in chromosome 2 (Table 3). The density of TE insertions into satellite arrays has been recently used to infer an age gradient for domains of such arrays around the primate X chromosome centromere [55].

**Table 3:** Nesting of repeats in human chromosomes Y and 2

	no. of elements^*a*^		% chromosome^*b*^		% nested^*c*^		insertions/Kb^*d*^	
	Y	2	Y	2	Y	2	Y	2
SINE	10068	119383	10%	12%	46%	51%	0.48	0.63

LINE	6207	65661	25%	21%	32%	39%	0.81	1.66

LTR	5200	38395	18%	8%	43%	52%	0.57	0.68

satellite	2144	222	5%	0%	4%	37%	0.05	0.18

DNA	1464	26886	2%	3%	35%	46%	1.12	0.88

RNA	55	778	0%	0%	54%	56%	0.36	0.26

repeats total	25157	251667	59%	45%	38%	48%	0.65	1.19

non-repetitive^‡^	-	-	41%	55%	-	-	1.33^†^	1.00

^*a*^Number of repetitive elements (models) in each group. ^*b*^Percentage of available (cytologically euchromatic) chromosome sequence. ^*c*^Percentage of elements in each group that are inserted into any other repetitive element. ^*d*^Density of repetitive element insertions (of any kind) per kilo base pairs of sequence within each group. ^‡^Non-repetitive sequence here means that similarity to known families of high complexity repeats was not detected, but it includes low-complexity repeats. ^†^Excludes satellite repeats arrayed in tandem. (Satellites considered are high-complexity repeats).

Another noteworthy nesting pattern in the re-annotation is the difference between LINEs on the one hand, and SINEs and LTR-elements on the other. On chromosome 2, LINEs harbour on average over twice the density of TE insertions (of any kind) per unit of LINE sequence than do SINEs per unit of SINE sequence or LTR-elements per unit of LTR-element sequence. In addition, on chromosome 2 (and on the Y) the proportion of SINE and LTR-element insertions nested into other repetitive elements is higher than that of LINEs (Table 3). Multiple factors such as age, base composition, and insertional biases may contribute to these differences, although automated analysis of such factors is beyond what is currently implemented in REANNOTATE.

#### Autosomal vs Y chromosome comparison of endogenous retroviral ages

**Figure 6:**
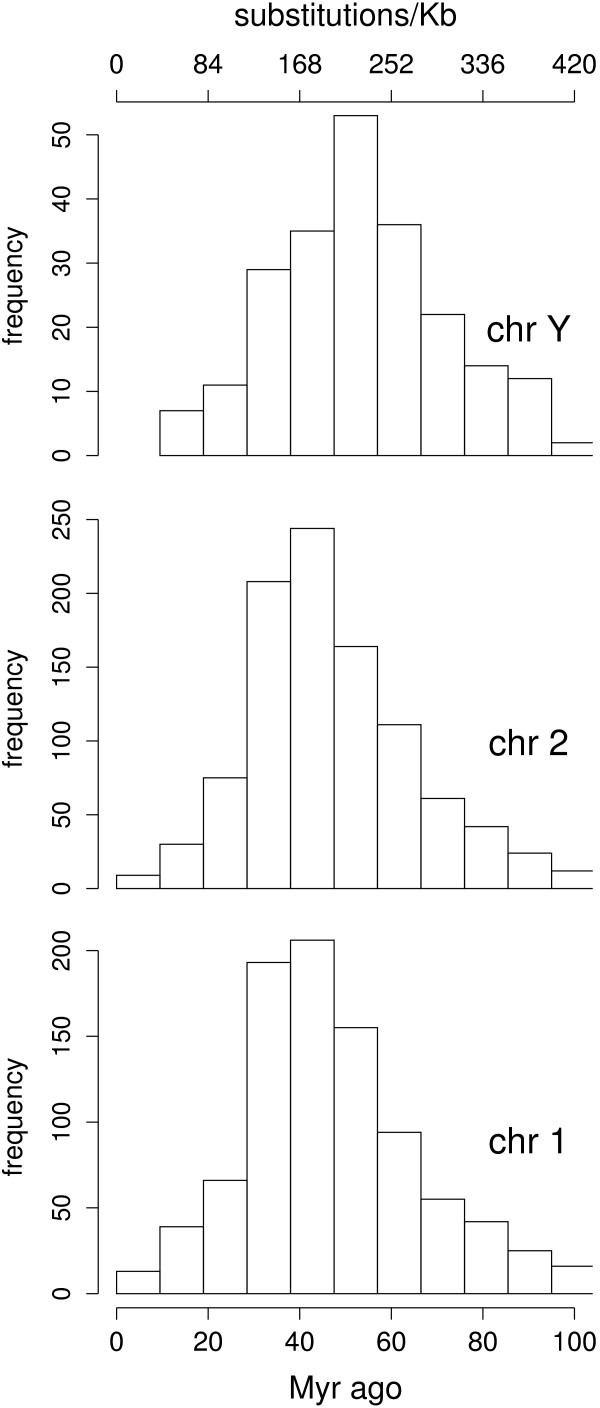
**Age distribution of endogenous retroviruses in the human genome**. Age distributions of endogenous retroviruses (ERVs) from the automated re-annotation of three human chromosomes. ERV intra-element LTRs on chromosome Y are significantly more divergent than those on chromosomes 2 and 1. Top axis shows the number of substitutions per kilo base pairs of intra-element LTR alignments. Age estimates obtained with a rate of 2.1 × 10^3 ^substitutions per site per million years [57].

The age distribution of endogenous retroviruses (ERVs) on human chromosomes 1, 2, and Y was obtained from their automated re-annotation, and shown in Figure 6. ERVs on both chromosomes 1 and 2 have less divergent intra-element LTRs than those on chromosome Y (Wilcoxon rank sum tests, *p *< 10^-6^), whilst there is no significant age difference between chromosomes 1 and 2 (*p *= 0.6). The main period(s) of retroviral activity over evolutionary time must have generated most ERV insertions on all chromosomes, therefore the older estimated ages are consistent with a faster rate of evolution on the Y than on chromosomes 1 and 2. Given that the "old" tail of the age distributions on those three chromosomes are similarly shaped, the age difference is not purely an effect due to longer persistence of ERVs on the Y. Evidence (from different methods) for a faster rate of evolution of the human Y chromosome relative to autosomes has been previously reported (e.g. [56]).

In contrast to plant (cereal) genomes where LTR-element polymorphisms make the dating of these elements potentially useful for dating haplotypes from different lines within the same species, most primate ERV insertions are ancient. Using the rate of evolution for mammalian repeats estimated in [57], 2.1 × 10^-3 ^substitutions per site per million years, the age distributions of ERVs in the genome of the lineage leading to human peaks at around 40 Myr ago.

### Discussion of potential applications

Re-annotation of repetitive elements via automated defragmentation, resolution of nesting structures, and (in some cases) dating of LTR-elements form the basis for the evolutionary analyses exemplified above. Re-annotation also makes possible other kinds of analyses that were not explored in this report. For example, *i) *analysis of the insertion sites of TEs, which would require proper defragmentation of fossil sequence fragments to identify the both termini of the ancestral TE sequence at the time of integration; *ii) *dating of LTR-elements could be useful for dating events on their host molecules, for instance when comparing haplotypes from cereal genomes where insertional polymorphism is common; and *iii) *analysis of global patterns of TE family nesting using network or interruption matrix analysis [24,32].

Defragmentation of repeats performed by REANNOTATE could also solve a problem that has plagued automated annotation of complex repeats: low-complexity regions *within *library sequences of reference high-complexity (dispersed) repeats, which results in low-complexity repeats in chromosomal sequence being annotated by REPEATMASKER as high-complexity repeats [22]. If these regions were masked on the reference sequences *prior *to their use in similarity searches, multiple hits might be reported even when the chromosomal sequence of a repetitive element is intact – this artefactual fragmentation is resolved by REANNOTATE into an element model, and masking of low-complexity regions in the reference library would be recommended in order to avoid low-complexity sequences being annotated as dispersed repeats. In addition to the re-annotation, the sequence output from REANNOTATE also has potential uses that were not explored in this report. Here this output was only utilised for dating LTR-elements, but alignment of all copies within a given family of repeats (which is non-trivial as copies typically have large indels relative to each other) is a powerful resource for evolutionary studies of repetitive elements. For instance, alignment of human ERV sequences (obtained with an early precursor to REANNOTATE) supported an analysis showing that members of the HERV-K family have been re-infecting the germline of the human lineage for 30 million years – from the inference of selective constraint on the HERV-K envelope gene [58]. Further examples of potential applications aided by multiple alignments of TE sequences are analyses of *i) *transition/transversion ratios in tests for the detection of hypermutability associated with cytosine methylation [59], *ii) *insertion/deletion spectra, used to estimate rates of spontaneous DNA loss [60], and *iii) *evolutionary relationships among individual elements or families via phylogeny re-construction [58,61].

### Limitations and scope for development

#### Reference library issues

As REANNOTATE takes as input similarity-based (REPEATMASKER) annotation, only elements homologous to known families of repeats can be re-annotated. For genomes with poorly characterised repeat families, any similarity-based detection methodology needs to be complemented with *de novo *repeat discovery [[62-65], for example]. Even though *de novo *discovery is useful for identifying uncharacterised families of repeats, it is not appropriate for genomic sequence annotation as it will fail to predict many repetitive elements that are are nested or fragmented. New families should be added to form an augmented reference library, and query sequences annotated with REPEATMASKER and REANNOTATE. Generally, sequence divergence between repetitive element lineages exposes the dependence of similarity-based annotation on the quality and comprehensiveness of the reference library. For defragmentation, the most difficult situation occurs when a chromosomal element is divergent from all available library elements. For example, if the chromosomal element is in parts homologous to two different library elements, then REannotate would construct two element models instead of one. Note that REannotate does provide a facility to correct defragmentation, provided that this situation is noticed through human inspection in the first place (see below).

Chimeric elements also present a challenging form of sequence divergence. They arise, for instance, when *i) *a transposable element nesting an unrelated element is mobilised, transducing the nested element; or *ii) *recombination between TE or ERV sequences leads to a new replication-competent element. If the progenitors of a chimeric element, but not the element itself, are both represented in the reference library, then REPEATMASKER will report separate hits to each progenitor reference, and REANNOTATE will construct separate element models for segments of the chimeric element. However, if upon human inspection of the automated annotation such chimerism is noticed, REANNOTATE does provide an option to combine hits to *prescribed *reference elements into element models, so that a new round of re-annotation will construct models capturing the full sequence of chimeric elements.

The optional REANNOTATE facility to defragment elements matching multiple reference sequences is also useful for solving another problem – *overrepresentation *of a lineage in the reference library, when the library redundantly contains very closely related sequences. Again this may cause REPEATMASKER to report matches to different reference elements that correspond to segments of the same chromosomal element. Finally, user-prescribed association of reference elements is essential for the construction of LTR-element models if the library entries for the LTR and IR representing the same family of elements have different names (as is the case for primate ERVs in REPBASE UPDATE).

However, the ideal approach for optimum similarity-based annotation is the construction of a reference library that non-redundantly represents repeat lineages as comprehensively as possible. In some cases, even when it is noticed (on human inspection) that chromosomal elements match multiple library sequences, the REannotate facility to defragment such elements will not help. This is the case when the rules for element model construction and defragmentation are violated. For example, the element labeled "3" in Table 1 (first column) and in Figure 4 was present in the original human annotation [51] with the name *Sabrina_F2-2*", an LTR-retrotransposon. The IR of this element was matched by three disntinct reference elements in the library used (Table 1: "SABRINA2_TM", "SABRINA3_TM", and "SABRINA_HV"). So in this case REannotate constructed three separate element models, which correspond to the element "*Sabrina_F2-2*" in the human annotation. The longest of these three models (labeled "3" in Figure 4) defragmented four hits to the reference element SABRINA2_TM (one IR and three LTR hits). The other two short element models are marked in Figure 4 by a horizontal bar and a "*". They correspond to the hits to SABRINA3_TM and SABRINA_HV. In the current defragmentation algorithm, these shorter hits could never be defragmented into the longer model containing the four SABRINA2_TM hits. This is because the SABRINA2_TM hits have in fact the opposite orientation on the chromosome relative to the SABRINA3_TM and SABRINA_HV hits.

#### DNA re-arrangements

REANNOTATE assembles fossil sequence fragments colinear with a given reference element into an element model, and the model assumes that such fragments are also colinear with the ancestral sequence of a repetitive element at the time of integration. DNA rearrangements involving a TE after its insertion into the genome may disrupt colinearity of its sequence with a reference element. The issue then arises as to whether to classify the re-arranged sequence as a single repetitive element. REANNOTATE will normally construct a separate model for any sequence segment violating colinearity with the reference. DNA rearrangements other than transposition (of an entire element) pose challenges for TE annotation; for example, if a segmental duplication has occurred within an element that remained replication-competent and that *subsequently *generated new copies, then for these new copies, colinearity with the reference element (which does not contain the duplication) is violated and two separate models are constructed. REANNOTATE does construct models of DNA rearrangements involving LTR-elements (which are based on re-arrangements of the common structure of these elements). One of the purposes of such annotation is cautioning against the validity of dating such an element, as the rearranged structure may be the result of inter-element recombination. The occurrence of post-integration, inter-element LTR recombination would invalidate the use of intra-element LTR sequence divergence as a molecular timer. It is also possible that inter-element LTR recombination may occur without altering the structure of the elements involved – in this case the re-arrangement will remain undetected by the algorithm currently implemented in REANNOTATE. Nevertheless, the aligned sequence data output by REANNOTATE can be used to reconstruct the phylogeny of all LTRs within a family: inter-element recombination would be detected if intra-element LTRs did not cluster on the phylogeny. There is scope for future implementation of algorithms for improving the detection and annotation of recombination, segmental duplication, and inversion events involving repetitive elements.

## Conclusion

REANNOTATE improves repetitive element annotation of genomic sequences by constructing models of evolutionary events involving dispersed repeats. Currently, automated repetitive element annotation is largely limited by default use of REPEATMASKER output, which reports genomic regions that have sequence similarity to known repeats. REANNOTATE is ready to post-process existing annotation or to be incorporated into annotation pipelines that use REPEATMASKER. REANNOTATE processes the similarity annotation to infer the common origin of dispersed repetitive sequences, resolve complex nesting patterns, and date insertion events LTR-elements with a detectable structure. These analyses become possible even in genomic regions with a high-density of repeats, such as heterochromatin. The annotation and repetitive element model sequences output by REANNOTATE therefore provide automated paleontology of complex repeats and, consequently, their host genomes, as the evolution (and possibly some function) of genomes is linked to their repetitive content.

## Availability and requirements

**Project name: **REannotate

**Project home page: **

**Operating system(s): **GNU/Linux or any other UNIX-like environment

**Programming language: **Perl

**License: **GNU GPL

**Any restrictions to use by non-academics: **None

The current version of REANNOTATE is also available as Additional file 5.

Usage, input, and output are described in the user manual, which is available as Additional file 6 and also at .

The programme CLUSTALW [39] or CLUSTALW2  is currently required for dating 'complete' LTR-elements.

## Abbreviations

ERV: endogenous retrovirus; HERV: human ERV; IR: internal region (of an LTR-element); LINE: long interspersed nuclear element; LTR: long terminal repeat; SINE: short interspersed nuclear element; TE: transposable element.

## Supplementary Material

Additional file 1 **Equivalence table of different reference sequences corresponding to the same families of ERVs.** Names of reference LTRs and internal regions of primate ERVs in REPBASE UPDATE that correspond to the same ERV family may be dissimilar. This text file contains lists (one per line) of equivalent (i.e. assigned to the same ERV family) names. This file must be input to REANNOTATE for defragmentation of primate ERVs if the input annotation was performed with the REPBASE UPDATE library. The first name in each line is used by REANNOTATE to assign a family to an element model that may contain hits matching different reference sequences within an equivalence class. Click here for file

Additional file 2 **Script for pre-processing primate ERV names in REPEATMASKER annotation**. A shell script for pre-processing REPEATMASKER annotation so that appropriate suffixes are added to the names of reference primate ERV internal regions (and LTRs). This is necessary for defragmentation of primate ERVs if the input annotation was performed with the REPBASE UPDATE library. Click here for file

Additional file 3 **Output re-annotation of dispersed repeats in the maize accession [GENBANK: **AF123535.1**].** This is the main repeat re-annotation file output by REANNOTATE as a tab delimited text file. Each line corresponds to a repetitive element model. Click here for file

Additional file 4 **Description of data fields in the main re-annotation file.** Data fields in Additional file 1. Click here for file

Additional file 5 **REANNOTATE.** Current version of REANNOTATE. Click here for file

Additional file 6 **REANNOTATE user manual.** User manual. Click here for file
