# Supplementary material for: Automated paleontology of repetitive DNA with REANNOTATE
Source: BMC Genomics. 2008 Dec 18;9:614. doi: 10.1186/1471-2164-9-614 (PMC2672092; doi:10.1186/1471-2164-9-614)
Supplement: Additional file 4 — Description of data fields in the main re-annotation file. Data fields in Additional file 1. [file 1471-2164-9-614-S4.pdf]

In the main annotation file output by REannotate each line corresponds to a defragmented repetitive element, apart from the first line, which is a header containing annotation field names. The annotation fields are described below:

<id> : REannotate identifier for the element.

<query> : Name of query sequence annotated to contain the element.

<family> : Name of reference library sequence most similar to identified element.

<div1> : (*LTR-elements only*). % divergence between each hit (if multiple hits, separated by hyphens) to the first LTR (along the query sequence) associated with the element and the library sequence.

<divI> : % divergence between each hit (separated by hyphens) to the element (or for LTR-elements this refers to hits to the internal region) and the library sequence.

<div2> : (*LTR-elements only*). % divergence between each hit (separated by hyphens) to the second LTR and the library sequence.

<start> : Start coordinate position in the query sequence of the (first hit to the) defragmented element.

<end1> : (*LTR-elements only*). End coordinate position in query of (last hit to) the first LTR.

<start2> : (*LTR-elements only*). Start coordinate position in query of (first hit to) second LTR.

<end> : End coordinate position in query of (last hit to) the defragmented element.

<hits1> : (*LTR-elements only*). Line numbers (if multiple separated by hyphens) on the RepeatMasker annotation file corresponding to hits to the first LTR.

<hitsI> : (*Excluding solo LTRs*). Line numbers (separated by hyphens) on the RepeatMasker annotation file corresponding to hits to the element (or for LTR-elements, hits to the internal region).

<hits2> : (*LTR-elements only*). Line numbers (separated by hyphens) on the RepeatMasker annotation file corresponding to hits to second LTR.

<nhits1> : (*LTR-elements only*). Number of hits identified as fragments of the first LTR.

<nhitsI> : (*Excluding solo LTRs*). Number of hits identified as fragments of the element (or for LTR-elements, fragments of the internal region).

<**nhits1**> : (*LTR-elements only*). Number of hits identified as fragments of the second LTR.

<**ref1**> : (*LTR-elements only*). Fraction of the reference sequence matched by (hits to) the first LTR.

<**refI**> : (*Excluding solo LTRs*). Fraction of the reference sequence matched by (hits to) the element (or for LTR-elements, hits to the internal region).

<**ref2**> : (*LTR-elements only*). Fraction of the reference sequence matched by (hits to) the second LTR.

<**lenR**> : Ratio of the span of the element on the query sequence (the length from the first to the last hit, including any intervening, unrelated insertions interrupting the element) to the length of the reference sequence.

<**orient**> : Orientation of the element on query sequence, + = forward, C = reverse.

<**superfamily**>**:** A larger evolutionary group into which the element family is classified.

<**nest**> : Level of nesting (into other repetitive elements) of this element; zero means the element is inserted into unique sequence (i.e. where no homology to known repeats has been detected); '1' means the element is inserted into another repeat, '2' means it is inserted into another repeat inserted into another repeat; and so on.

<**nestIDs**> : Hyphenated (if necessary) list of IDs of other elements into which this element is nested.

<**DNArearrangement**> : (*LTR-elements only*). REANNOTATE detects certain kinds of DNA rearrangements involving LTR-elements, other than transposition of the whole element. If such a rearrangement is detected, a hyphenated list of the line numbers on the REPEATMASKER annotation file corresponding to the hits involved is given. (If the putative rearrangement involves only the internal region, the list is marked with a "*".)

<**K**> : Estimate of the number of nucleotide substitutions per site between the LTR pair of a structurally 'complete' LTR-element (using the Kimura 2-parameter model). If a different kind of element is either nested in, or nesting, a complete LTR-element, the equivalent of an upper or lower bound is given.

<**K.sd**> : Standard deviation of the estimate of <**K**> between intra-element LTRs.

<**time**> : Estimate of the time (in million years ago) since the chromosomal integration of a 'complete' LTR-element ($t = \frac{K}{2r}$, where r is a rate of evolution supplied by the user). If a different kind of element is either

nested in, or nesting, a complete LTR-element, an upper or lower bound is given.

<**time.sd**> : Standard deviation for the estimate of <**time**>, including stochasticity in the accumulation of nucleotide substitutions.

<**numSites**> : (*'Complete' LTR-elements only*). Number of nucleotide sites aligned between intra-element LTRs.

<**T**> : (*'Complete' LTR-elements only*). Number of nucleotide transitions per site between intra-element LTRs.

<**V**> : (*'Complete' LTR-elements only*). Number of nucleotide transversions per site between intra-element LTRs.
