# Supplementary material for: Automated paleontology of repetitive DNA with REANNOTATE
Source: BMC Genomics. 2008 Dec 18;9:614. doi: 10.1186/1471-2164-9-614 (PMC2672092; doi:10.1186/1471-2164-9-614)
Supplement: Additional file 6 — REANNOTATE user manual. User manual. [file 1471-2164-9-614-S6.pdf]

REannotate
User Manual

Contents

1 INPUT **1**
1.1 Required command line argument 1
1.2 Command line argument required for *sequence output* 2
1.3 Command line argument required for joint defragmentation of matches to multiple reference elements 2

2 USAGE **2**
2.1 Command line usage 2
2.2 Command line OPTIONS 3
2.3 Example 4

3 OUTPUT **4**
3.1 Re-annotation 5
3.1.1 Main annotation (Defragmentation, Nesting, Time) . . . 5
3.1.2 Re-annotation of RepeatMasker IDs 7
3.1.3 General Feature Format (GFF) output and visualisation in Apollo 8
3.2 Sequences 9
3.2.1 Output directories 9
3.2.2 File naming scheme and sequence orientation 9
3.2.3 Alignments 10

1 INPUT

1.1 Required command line argument

REannotate takes a RepeatMasker (http://www.repeatmasker.org) annotation (.out) file as input, or alternatively a RepeatMasker table downloaded from the UCSC (University of California at Santa Cruz) Genome Browser web site (http://genome.ucsc.edu). See usage in section 2 below.

1.2 Command line argument required for *sequence output*

If the DNA sequence of repetitive elements defragmented by REannotate is required, then the genomic sequences annotated with RepeatMasker (whose annotation is input 1.1) must be input in FASTA format to REannotate. See usage in section 2 below.

1.3 Command line argument required for joint defragmentation of matches to multiple reference elements

If matches to multiple reference elements are to be considered for defragmentation (see the scientific paper), a text file with lists (one per line) of "equivalent" reference element names must be supplied. See usage in section 2 below. For example, a file containing the following two lines

```
# equivalent Zea mays repeat family names
cinful1 cinful2 cinful1_zm cinful2_zm
```

would inform REannotate to consider matches to reference elements named either cinful1 or cinful2 or cinful1_zm or cinful2_zm for defragmentation into a repetitive element model. Lines starting with '#' are comments.

2 USAGE

REannotate is a Perl script that runs on GNU/Linux and other UNIX-like operating systems/environments. You need to run it from a directory to which you have permission to write.

2.1 Command line usage

REannotate is a command line tool with the following USAGE:

```
: (perl) REannotate [ -OPTIONS ] <RepeatMasker annotation file> [ sequence file ]
```

The filename (if in current directory) or path to the <RepeatMasker annotation file> is a required argument.
The filename or path to the [sequence file] argument is optional; if no sequence output is required then the [sequence file] argument is omitted and OPTION '-n' must be used:

```
: REannotate -n [ -OPTIONS ] <RepeatMasker annotation file>
```

2.2 Command line OPTIONS

-h (-help) : Displays command line options

-v (-version) : Displays version of REannotate

-n (-noseq) : Suppresses all sequence output, so that REannotate will only generate annotation. If this option is used no sequence input is required.

-u (-ungapped) : This option outputs the chromosomal sequence *spanned* by a defragmented element (from the first to the last fragment), in addition to the default gapped element sequence that is aligned to a reference element.

-k (-kalign) <path to clustalW> : Aligns intra-element LTR pairs using the clustalW program and estimates K (the number of nucleotide substitutions between intra-element LTRs) using the Kimura 2-parameter model, for each complete element found. Required argument is the path to the clustalW directory. (Option **-n** must not be used).

-r (-rate) <rate of evolution> : Estimates the time elapsed since the insertion of structurally 'complete' LTR-elements, if the **-k** option is being used. The required argument is the rate of evolution in number of substitutions per site per million years.

-g (-gff) : Outputs extra annotation in GFF format (e.g. for visualisation in the Apollo Genome Browser; see section 3 below).

-f (-fuzzy) <filename> : Allows defragmentation of chromosomal repeat fragments that match different reference elements. The required argument is the (path to) filename of a text file containing lists of 'related' (equivalent) reference sequence names (one per line; see the scientific paper for details).

-t (-tnest) : Allows 'truncated nesting' to be annotated, i.e. elements that interrupt one terminus of another element (but which are not contained within the latter) will be annotated as nested. (See Methods in the scientific paper).

-d (-drange) <distance in nucleotides> : Sets the 'search range' for defragmentation, the maximum distance (in number of nucleotides) that is allowed between two repeat fragments to be considered as a candidate fragments of the same element. (This parameter is called δ in the Methods of the scientific paper). Required argument is the search range in nucleotides. Default = 40000.

-b (-boundary) <distance in nucleotides> : Sets the maximum tolerated overlap (in number of nucleotides along a reference element sequence) between chromosomal matches to the same reference sequence to be considered as fragments of the same chromosomal element. (This parameter is called ϵ in the Methods of the scientific paper). Required argument is the alignment overlap in nucleotides along a reference sequence. Default = 40.

-s (-solo) <distance in nucleotides> : Sets the minimum distance required (in number of nucleotides) between a given LTR and any other LTR or internal region (belonging to the same family and in the same orientation on the host chromosome) for the LTR to be considered as a 'solo' LTR. (This parameter is called σ in the Methods of the scientific paper). Required argument is the distance in nucleotides. Default = 15000.

-m (-stream) : Outputs re-annotation to STDOUT (all messages saved in file <REannotate_output/re.messages>)

-l (-long) : Outputs ASCII representation of the order and nesting of defragmented elements along a query sequence. (NOT RECOMMENDED FOR LARGE QUERIES!)

-c (-class) : Outputs re-annotation to four separate files (instead of the one file by default), one file for each class: non-LTR-elements, complete LTR-elements, truncated LTR-elements, and solo LTRs.

2.3 Example

For example, from the command line below

```
: REannotate -k /usr/bin/clustalw -r 0.015 query.out query.fasta
```

REannotate will invoke clustalW to align (defragmented) intra-element LTRs and estimate the number of substitutions that have occurred between them, and then estimate the time since element integration using a rate of evolution equal to 0.015 substitutions per site per million years. (This is in addition to the default defragmentation and analysis of the nesting structure of all complex repeat fragments in the query sequence that have been annotated by RepeatMasker). REannotate will output the sequence of all defragmented elements as default, and with the above options it will also output the clustalW alignments of defragmented intra-element LTRs.

3 OUTPUT

Inside the directory from which it is run, REannotate creates a sub-directory called

```
REannotate_output
```

to contain all annotation and sequence output.

3.1 Re-annotation

3.1.1 Main annotation (Defragmentation, Nesting, Time)

The main annotation is output to a single tab-delimited text file with the ".RE-annotation" extension:

```
REannotate_output/<prefix>.REannotation
```

Alternatively, if option **-c (-class)** is used then four files with extension ".REannotation" will be generated

```
REannotate_output/non-LTR_elements/nonLTR.REannotation
REannotate_output/LTR_elements/complete.REannotation
REannotate_output/LTR_elements/truncated.REannotation
REannotate_output/LTR_elements/solos.REannotation
```

so that the annotation is output separately for non-LTR elements, structurally complete LTR-elements, truncated LTR-elements, and 'solo' LTRs, respectively.

In the above annotation files each line corresponds to a defragmented repetitive element (apart from the first line, which is a header containing annotation field names), and the columns contain the following annotation fields:

<id> : REannotate identifier for the element.

<query> : Name of query sequence annotated to contain the element.

<family> : Name of reference library sequence most similar to identified element.

<div1> : (*LTR-elements only*). % divergence between each hit (if multiple hits, separated by hyphens) to the first LTR (along the query sequence) associated with the element and the library sequence.

<divI> : % divergence between each hit (separated by hyphens) to the element (or for LTR-elements this refers to hits to the internal region) and the library sequence.

<div2> : (*LTR-elements only*). % divergence between each hit (separated by hyphens) to the second LTR and the library sequence.

<start> : Start coordinate position in the query sequence of the (first hit to the) defragmented element.

<end1> : (*LTR-elements only*). End coordinate position in query of (last hit to) the first LTR.

<**start2**> : (*LTR-elements only*). Start coordinate position in query of (first hit to) second LTR.

<**end**> : End coordinate position in query of (last hit to) the defragmented element.

<**hits1**> : (*LTR-elements only*). Line numbers (if multiple separated by hyphens) on the RepeatMasker annotation file corresponding to hits to the first LTR.

<**hitsI**> : (*Excluding solo LTRs*). Line numbers (separated by hyphens) on the RepeatMasker annotation file corresponding to hits to the element (or for LTR-elements, hits to the internal region).

<**hits2**> : (*LTR-elements only*). Line numbers (separated by hyphens) on the RepeatMasker annotation file corresponding to hits to second LTR.

<**nhits1**> : (*LTR-elements only*). Number of hits identified as fragments of the first LTR.

<**nhitsI**> : (*Excluding solo LTRs*). Number of hits identified as fragments of the element (or for LTR-elements, fragments of the internal region).

<**nhits1**> : (*LTR-elements only*). Number of hits identified as fragments of the second LTR.

<**ref1**> : (*LTR-elements only*). Fraction of the reference sequence matched by (hits to) the first LTR.

<**refI**> : (*Excluding solo LTRs*). Fraction of the reference sequence matched by (hits to) the element (or for LTR-elements, hits to the internal region).

<**ref2**> : (*LTR-elements only*). Fraction of the reference sequence matched by (hits to) the second LTR.

<**lenR**> : Ratio of the span of the element on the query sequence (the length from the first to the last hit, including any intervening, unrelated insertions interrupting the element) to the length of the reference sequence.

<**orient**> : Orientation of the element on query sequence, + = forward, C = reverse.

<**superfamily**>**:** A larger evolutionary group into which the element family is classified.

<**nest**> : Level of nesting (into other repetitive elements) of this element; zero means the element is inserted into unique sequence (i.e. where no homology to known repeats has been detected); '1' means the element is inserted into another repeat, '2' means it is inserted into another repeat inserted into another repeat; and so on.

<nestIDs> : Hyphenated (if necessary) list of IDs of other elements into which this element is nested.

<DNArearrangement> : (*LTR-elements only*). REannotate detects certain kinds of DNA rearrangements involving LTR-elements, other than transposition of the whole element. If such a rearrangement is detected, a hyphenated list of the line numbers on the RepeatMasker annotation file corresponding to the hits involved is given. (If the putative rearrangement involves only the internal region, the list is marked with a "*".)

<K> : Estimate of the number of nucleotide substitutions per site between the LTR pair of a structurally 'complete' LTR-element (using the Kimura 2-parameter model). If a different kind of element is either nested in, or nesting, a complete LTR-element, the equivalent of an upper or lower bound is given.

<K.sd> : Standard deviation of the estimate of **<K>** between intra-element LTRs.

<time> : Estimate of the time (in million years ago) since the chromosomal integration of a 'complete' LTR-element ($t = \frac{K}{2r}$, where r is a rate of evolution supplied by the user). If a different kind of element is either nested in, or nesting, a complete LTR-element, an upper or lower bound is given.

<time.sd> : Standard deviation for the estimate of **<time>**, including stochasticity in the accumulation of nucleotide substitutions.

<numSites> : (*'Complete' LTR-elements only*). Number of nucleotide sites aligned between intra-element LTRs.

<T> : (*'Complete' LTR-elements only*). Number of nucleotide transitions per site between intra-element LTRs.

<V> : (*'Complete' LTR-elements only*). Number of nucleotide transversions per site between intra-element LTRs.

3.1.2 Re-annotation of RepeatMasker IDs

In addition to the main annotation output (the file with extension ".REannotation", section 3.1.1), REannotate outputs a copy of the input RepeatMasker file — but with the original ID column replaced by defragmented element IDs corresponding those in the main REannotate annotation file. This modified copy of the input annotation is output to a file with the ".REannotated" extension:

```
REannotate_output/<prefix>.REannotated
```

3.1.3 General Feature Format (GFF) output and visualisation in Apollo

Invoking REannotate with the **-g|gff** option

```
: REannotate -g [ -OPTIONS ] <RepeatMasker annotation file> [ sequence file ]
```

will produce two GFF annotation files:

```
REannotate_output/<prefix>.REannotate.gff
REannotate_output/+<prefix>.REannotate.gff
```

The GFF file with a "+" symbol on its name is output for visualisation of REannotate's defragmentation and nesting annotation in the Apollo genome browser. This a variation on the conventional GFF format, only because the column that conventionally informs on the orientation of a feature on a given chromosome, here is set to "+" (forward strand) for all defragmented repetitive elements — for the purpose of visualising their nesting structure in Apollo. The orientation of the elements is nevertheless indicated in the first column containing element IDs, which in this GFF file have the format: "<ID><orientation>_<reference family>", where <ID> corresponds to a defragmented element ID in the main annotation (section 3.1.1), and <orientation> is either "+" or "-". So for example, the GFF ID:

u1-_OPIE2_ZM

indicates a defragmented transposable element of the family *OPIE2_ZM* inserted on the reverse strand, whose ID in the main REannotate annotation file is *u1*.

The other GFF file (whose name is output without the "+") has the same IDs but a conventional orientation column. (This makes visualisation of the nesting structure more difficult in Apollo).

Finally, in order to visualise REannotate's defragmentation and nesting analyses in Apollo, the contents of the file

```
<REannotate.tiers>
```

(which is distributed with REannotate) must be appended to the the file <Enseml.tiers>, which is part of the Apollo distribution. (Alternatively, if REannotate's annotation will not be combined with other sources, the original <Ensembl.tiers> may be moved to a different name, and then <REannote.tiers> renamed <Ensembl.tiers>).

3.2 Sequences

REannotate outputs the DNA sequence of each defragmented element model. These sequences locally aligned to the closest matching reference sequence (see the scientific paper). (If option **-u|ungapped** is used, then the entire chromosomal sequence from the first to the last fragment assembled into an element model is output, instead).

3.2.1 Output directories

Non-LTR element sequences are output to directory

```
REannotate_output/non-LTR_elements/
```

and LTR-element sequences are output to three separate directories

```
REannotate_output/LTR_elements/complete/<subdirectory>/
REannotate_output/LTR_elements/truncated/<subdirectory>/
REannotate_output/LTR_elements/solo/
```

for structurally 'complete' (i.e. elements retaining at least part of both their LTRs), truncated, and solo LTR-elements, respectively. The 'complete' and 'truncated' element sequences are further organised into three subdirectories named "full", "internal", and "LTRs" (these respectively contain the sequences of the full LTR-element models, the internal region models, and the LTR models).

3.2.2 File naming scheme and sequence orientation

Sequence files are named according to the following scheme

```
<ID>_<query sequence>_<orientation>_<reference family><suffix>.fasta

( <ID>_<query sequence>_<orientation>_<reference family><suffix>.ungapped.fasta )
```

where <ID> corresponds to a defragmented element ID in the main annotation (section 3.1.1), <query sequence> is the name of the query sequence containing the element, and <orientation> of the element is either "+" or "C" (forward or reverse strand). A further <suffix> may be appended to the file names in some cases.

Note that sequences in the reverse orientation ("C") need to be reverse-complemented if they are to be subsequently aligned to other sequences of the same reference family in the forward ("+") orientation.

3.2.3 Alignments

If option **-k|kalign** is used, then CLUSTALW alignments of intra-element LTR pairs are saved in directory

```
REannotate_output/LTR_elements/complete/LTRs/alignments/
```
